# Supplementary material for: Genopo: a nanopore sequencing analysis toolkit for portable Android devices
Source: Commun Biol. 2020 Sep 29;3:538. doi: 10.1038/s42003-020-01270-z (PMC7524736; doi:10.1038/s42003-020-01270-z)
Supplement: Supplementary file 1 — Supplementary Information [file 42003_2020_1270_MOESM1_ESM.pdf]

SUPPLEMENTARY MATERIALS

Genopo: A nanopore sequencing analysis toolkit for portable Android devices

Hiruna Samarakoon[1], Sanoj Punchihewa[1], Anjana Senanayake[1], Jillian M. Hammond[2], Igor Stevanovski[2], James M. Ferguson[2], Roshan Ragel[1], Hasindu Gamaarachchi[2,3]* & Ira W. Deveson[2,4]*

[1] Department of Computer Engineering, University of Peradeniya, Peradeniya, Sri Lanka

[2] Kinghorn Centre for Clinical Genomics, Garvan Institute of Medical Research, Sydney, Australia

[3] School of Computer Science and Engineering, University of New South Wales, Sydney, Australia

[4] St Vincent's Clinical School, University of New South Wales, Sydney, Australia

* Contributed equally - correspondence: hasindu@garvan.org.au, i.deveson@garvan.org.au

Supplementary Table 1 p.2

Supplementary Table 2 p.3

Supplementary Table 3 p.4

Supplementary Note 1.1 p.5

Supplementary Figure 1 p.5

Supplementary Note 1.2 p.7

Supplementary Figure 2 p.7

Supplementary Note 2 p.8

Supplementary Figure 3 p.8

Supplementary Figure 4 p.9

Supplementary references p.10

Supplementary Data 1 [provided as separate Excel sheet]

Detailed run-time information for SARS-CoV-2 genome analysis.

Supplementary Data 2 [provided as separate Excel sheet]

Detailed run-time information for NA12878 methylation calling analysis.

Sample	Reads	Library size (Mbases)	Mean genome coverage (x-fold)	Workflow run-time (minutes)				
				A - Huawei	B - Nokia	C - LG	D - Sony	H - HPC
SARS-CoV-2_001	4016	10.5	316	21.91	9.30	27.57	38.36	1.55
SARS-CoV-2_002	4701	12.3	358	28.61	10.75	39.76	26.35	1.75
SARS-CoV-2_003	4438	11.6	340	21.82	13.10	27.37	21.36	1.47
SARS-CoV-2_004	4984	13.0	384	26.44	11.15	32.47	51.08	1.65
SARS-CoV-2_005	4128	10.8	318	25.77	10.93	32.92	59.80	1.47
SARS-CoV-2_006	4148	10.8	323	21.61	8.00	27.32	18.36	1.30
SARS-CoV-2_007	3823	10.8	300	34.87	12.91	46.77	26.00	1.55
SARS-CoV-2_008	4556	11.9	353	29.41	10.65	35.44	25.93	1.50
SARS-CoV-2_009	4230	11.1	322	23.11	10.81	36.04	64.11	1.77

Supplementary Table 1. Run-time comparison of four smartphone models (A-D) for SARS-CoV-2 genome sequencing analysis. Device H is a high performance server computer (Dell PowerEdge R740xd) – HPC jobs were run with 384 GB RAM and 32 cores available.

Device	Manufacturer	Model	RAM (GB)	Internal storage (GB)	Cores	Clock speed (Ghz)	CPU architecture	Kernel OS
A	Huawei	AMN LX2	2	32	4	2	aarch64	armeabi-v7a
B	Nokia	6.1 Plus	4	64	8	1.4-1.8	ARMv8	arm64-v8a
C	LG	Q6	3	32	8	1.4	ARMv8	armeabi-v7a
D	Sony	XA Ultra	3	16	8	4x2-4x1	aarch64	arm64-v8a
E	Huawei	Nova 5T	8	128	8	1.46-2.6	ARMv8	arm64-v8a
F	Samsung	A70	8	120	8	2x2-6x1.7	ARMv8	arm64-v8a
G	Xiaomi	Mi 9T	6	120	8	2.2	ARMv8	arm64-v8a

Supplementary Table 2. Technical specifications for Android smartphones used in the study.

Sample	Flow-cell ID	Reads	Library size (Mbases)	Run-time (minutes)				
				B - Nokia	E - Huawei	F - Samsung	G - Xiaomi	H - HPC
Complete run	FAB42804	16,688	91	20.40	21.48	22.68	20.18	1.89
Single batch	FAF05869	4,000 (repeated)	34	16.58	13.18	14.25	12.34	1.53

Supplementary Table 3. Run-time comparison of four smartphone models for methylation profiling analysis with NA12878 sequencing data. Device H is a high performance server computer (Dell PowerEdge R740xd) – HPC jobs were run with 384 GB RAM and 32 cores available.

Supplementary Note 1: Bioinformatics workflows executed with *Genopo*.

1.1. ARTIC pipeline for SARS-CoV-2 genome analysis

The ARTIC network for viral surveillance has established a standardised bioinformatics pipeline for SARS-CoV-2 genome analysis with ONT sequencing data (https://github.com/artic-network/artic-ncov2019). This workflow has been integrated into *Genopo* for the analysis of SARS-CoV-2 patient isolates and is summarised in **Fig. S1**. While this workflow is specifically tailored for analysis of SARS-CoV-2, *Genopo* also supports a generic variant calling pipeline (comprised of *Minimap2*[1], *Samtools*[2] & *Nanopolish*[3]) that can be used to detect variants in any organism where a reference genome is available.

Base-called reads (.fastq) are first aligned to the SARS-CoV-2 reference genome (MN908947) using *Minimap2* then sorted and indexed using *Samtools* (v1.10). Primer sites are trimmed from alignments and coverage normalisation performed using *Artic_c trim* (v1.0.0), a C/C++ re-implementation of the original *aligntrim.py* Python script in the ARTIC repository. Trimmed and normalised alignments are again sorted and indexed using *Samtools*. ONT raw signal files (.fast5) are indexed using *Nanopolish index*, then variant calling is performed using *Nanopolish variants* (v0.11.3). Note that depending on the available RAM on the device, the variant calling step may have to be run multiple times while iterating through smaller genomic windows at a time (-w option). This is a common practice even on high-performance computers when the genome is large. The more RAM a device has, the larger the window size can be.

After obtaining the variant calls in VCF format, the user can optionally proceed to build a consensus genome for their SARS-CoV-2 isolate, using *Bcftools* (v1.10.2)[2]. VCF files generated from *Nanopolish* lack meta-information in the header to be compatible with *Bcftools*. Therefore, the VCF header is first modified using *Bcftools reheader*. Reads with low coverage are then identified using *Samtools* depth followed by *Artic_c mask*, a sub-tool that generates a BED file specifying low-coverage regions of the genome (< 20-fold). Variants with low quality scores (QUAL < 200) are identified using *Bcftools query* and concatenated with the low coverage BED file and another BED file containing explicitly specified (pre-defined) ambiguous bases to produce a single BED file for masking. BED file concatenation is performed using our sub-tool *Artic_multiinter*, which is functionally equivalent to *Bedtools multiinter*[4]. Finally, the consensus genome is built using *Bcftools consensus* where the ambiguous bases in the masking BED file are replaced with 'N'.

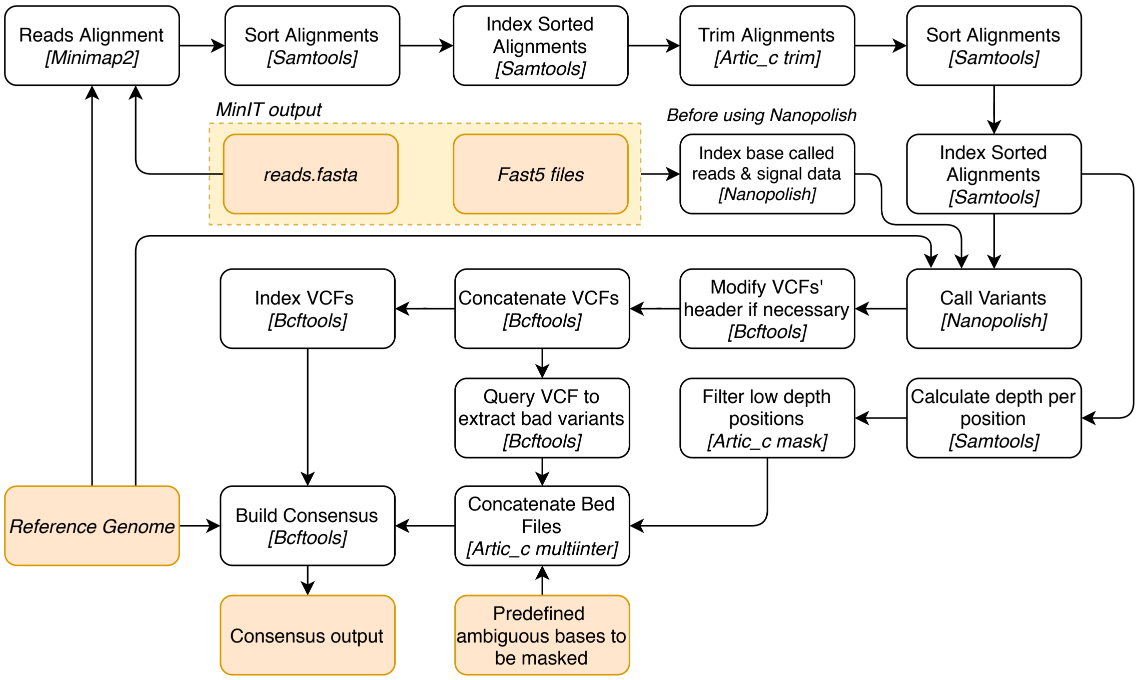

Fig. S1. Schematic summary of SARS-CoV-2 genome analysis workflow executed with Genopo.

Complete commands are as follows:

1) Aligning reads to the virus genome using *Minimap2*

```
minimap2 -x map-ont [reference] [reads.fastq|fasta] -o [output.sam file] -a -t [no. of threads [4]] -K [Number of bases loaded into memory to process in a mini-batch [5M]]
```

2) Sort aligned reads using *Samtools*

```
samtools sort [minimap2 output] -o [output.bam file]
```

3) Index sorted reads using *Samtools*

```
samtools index [samtools sort output]
```

4) Trim (downsize) alignments using *Artic_c*

```
artic_c trim -n [normalise threshold [200]] -b [primer info bed file] -i [samtools sort output] -o [output .bam file]
```

5) Sort trimmed reads using *Samtools*

```
samtools sort [articc output] -o [output .bam file]
```

6) Index sorted reads using *Samtools*

```
samtools index [samtools sort output]
```

7) Find depth (coverage) of positions using *Samtools*

```
samtools depth -a [samtools index output] -o [output .bed file]
```

8) Create bed file containing low depth (coverage) positions

```
artic_c mask -d [depth threshold [20]] -i [samtools depth output] -o [.bed file containing low depth positions]
```

9) Index fast5 files using *Nanopolish* (run once only for all datasets)

```
nanopolish index --directory [fast5folder] [reads.fastq|fasta]
```

10) DNA variant calling using *Nanoplish* (run multiple times for adjacent windows if device's RAM is not enough)

```
nanopolish variants -r [reads.fastq|fasta] -b [samtools index output] -g [reference] -o [output.vcf file] -t [no. of threads [8]] -w [windowsize(<chromosomename>:<start>-<end>)] -m [variant frequency [0.1]] -d [depth[20]] -x [number of haplotypes combinations [1000000]] -p [ploidy level[1]] --snps[flag to call only SNPs] --fix-homopolymers [flag to run homopolymer caller]
```

11) Change header using *Bcftools* (repeat for each *nanopolish variant* output)]

```
bcftools reheader -f [.fai reference file] [vcf file] -o [output.vcf file]
```

12) Concatenate VCF files using *Bcftools*

```
bcftools concat [list of vcf files from step 11] -O [output format [b]] -o [output.bcf file]
```

13) Index VCF file using *Bcftools*

```
bcftools concat [bcftools view output]
```

14) Query concatenated vcf file to filter low quality variants into a bed file using *Bcftools*

```
bcftools query -i [filter condition ['QUAL<200 || INFO/TotalReads<20']] -f [fileformat ['%CHROM\t%POS0\t%END\t%ID\n']] [bcftools concat output] -o [output.bed file]
```

15) Concatenate bed files containing masking bases using *artic_c*

```
articc multiinter -i [bcftools query output] [articc mask output] [predefinedbases to be masked] -o [output.bed file]
```

16) Generate consensus using *Bcftools*

```
bcftools consensus -m [artic_c multiinter output] -f [reference] [bcftools concatoutput] -o [output.fasta file]
```

1.2. Methylation profiling in NA12878

Genopo currently supports complete pipelines for methylation calling and event alignment. The methylation calling pipeline is illustrated in this article and summarised in **Fig. S2**. The pipeline's inputs are a reference genome (e.g., hg38), ONT raw signal files (.fast5) and corresponding base-called reads (.fastq). Base-called reads are first aligned to the reference genome using *Minimap2* (v2.17)[1]. The memory usage for *Minimap2* increases with the size of the reference genome, causing failures for large reference genomes when running on mobile devices with limited memory. To overcome this constraint, we used an index partitioning strategy described elsewhere[5], integrating this algorithm into our pipeline along with *Minimap2*. Note that the reference partitioning should be done on a computer, before storing the partitioned reference on the smartphone (see Advanced Usage Instructions). The aligned reads are then sorted and indexed using Samtools (v1.10)[2]. Read polishing is then performed using *Nanopolish*[3]. We adopt a re-engineered version of Nanopolish called *f5c*[6], which is both memory and time efficient. *F5c* first indexes base-called reads and raw signal data. Subsequently, *f5c* can either perform methylation calling or event alignment, at the user's request.

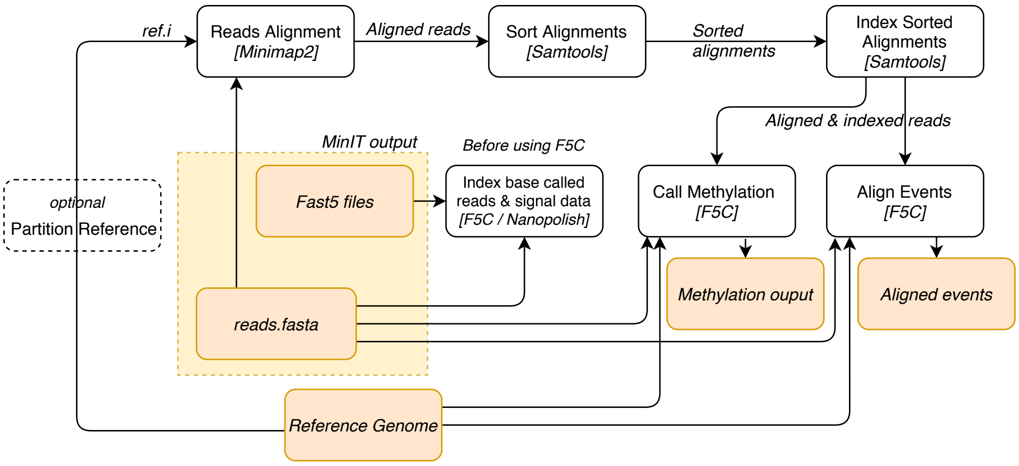

Fig. S2. Schematic summary of methylation profiling workflow executed with Genopo.

Complete commands are as follows:

1) Partitioning the reference genome (on a computer)

```
divideandindex.sh [reference.fa file] [numparts [8]] [output.idx file][minimap2exe] [minimap2profile [map-ont]]
```

2) Aligning reads to the partitioned human genome using *Minimap2*

```
minimap2 -x map-ont --split-prefix [temporary file path] [partitioned reference][reads.fastq|fasta] -o [output.sam file] -a -t [no. of threads [4]] -K [Number of bases loaded into memory to process in a mini-batch [5M]]
```

3) Sort aligned reads using *Samtools*

```
samtools sort [minimap2 output] -o [output.bam file]
```

4) Index sorted reads using *Samtools*

```
samtools index [samtools sort output]
```

5) Index fast5 files

```
f5c index --directory [fast5folder] [reads.fastq|fasta]
```

6) DNA methylation calling using *f5c*

```
f5c call-methylation -r [reads.fastq|fasta] -b [path to samtools index output] -g [ref.fa] -o [output meth.tsv] -B [max number of bases loaded at once [2.0M]] -K [max number of reads loaded at once [256]]
```

7) Aligning nanopore events to reference k-mers using *f5c* (optional, not a step in methylation calling)

```
f5c eventalign -r [reads.fastq|fasta] -b [path to samtools index output] -g [ref.fa] -o [output meth.tsv] -B [max number of bases loaded at once [2.0M]] -K [max number of reads loaded at once [256]] --summary [output events.summary.txt]
```

Supplementary Note 2: *Genopo* user guide

2.1. Basic usage information

Genopo has four major functionalities, which are listed below and shown in **Fig. S4a**.

1) Stand-alone mode for configuration and execution of a custom pipeline on the mobile device.

2) Mobile-cluster mode for real time analysis using a cluster of mobile devices, which is currently under development and will not be discussed in this paper.

3) Download data-sets using URLs and extract compressed files.

4) An example demonstration of downloading and extracting a nanopore data-set of E. Coli Bacteria, followed by executing a complete methylation calling pipeline on the data-set.

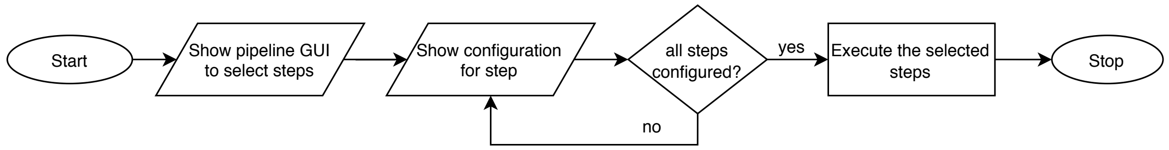

Fig S3. Schematic summary of basic Genopo stand-alone usage.

Genopo includes a 'help' section for new users to get started. It has a summary of the above four major functionalities (**Fig. S4b**). Once *Genopo* mobile application is launched for the first time, the user is prompted to grant permission to read and write from the internal storage of the mobile device. In most of the devices, once this permission is set, it is adequate to read from and write to the external storage (SD card) as well. However, in certain devices the user is expected to set this permission explicitly, which can be done by navigating to [help→set SD card permission] section (**Fig. S4c**). *Genopo*'s start page has listed down the above four functionalities (**Fig. S4a**). A user navigating to stand-alone mode will land on a page to select custom pipeline steps from where he can choose Minimap2, Samtools, *f5c* or a desired combination of those tools (**Fig. S4f**). Once the steps are selected, the user can choose either GUI mode or (**Fig. S4g,h**) to configure parameters for each tool. **Fig. S3** shows the procedure to use stand-alone mode. It is recommended to use the GUI mode as the final commands are always compiled into a set of strings and later shown in the terminal mode before proceeding to the execution. In GUI mode file path arguments get auto completed once the user set the correct path to the data set directory. *Genopo* provides an elegant directory navigator for this purpose and both GUI mode and terminal mode have it. If the user chose terminal mode at the beginning, he skips GUI mode and lands on the terminal mode. From the terminal mode the user can proceed to the pipeline execution page (**Fig. S4i**). Once the pipeline execution is started a timer will be displayed. After the execution of the pipeline the user can write results to a log file (named f5n.log) which is located inside storage/mobile-genomics directory. In the rare event of a crash of *Genopo*, the user can run the previous pipeline using LOAD PREVIOUS CONFIGURATION command. If the app crashes during an execution, the user can identify the error occurred by referring tmp.log which is located inside storage/mobile-genomics folder. For more information regarding log files please refer the help section on home page in the application. Functionalities to download a data-set form a URL and extract a compressed data-set are available on a same page (**Fig. S4d**). To download a data-set, the user has to set the specific data-set URL path and the location on the storage to where the data-set should be downloaded. Decompressing a file is as easy as setting the file path of the compressed file and pressing the EXTRACT button. The decompressed file will have the same location as the compressed file. Since a data-set usually consists of many numbers of considerably small fast5 files, it will take much time to transfer them to a device storage unless the files are compressed. Hence, *Genopo* is provided with a file extraction functionality to decompress the files as necessary. In mobile-cluster mode compressed files will get transferred over WiFi. The example demonstration is a setup with only three steps to help users get familiar with *Genopo*. The steps involve the basic procedure to execute a pipeline. They are 1, download a data set 2, extract the data set and 3, execute the pipeline (**Fig. S4e**).

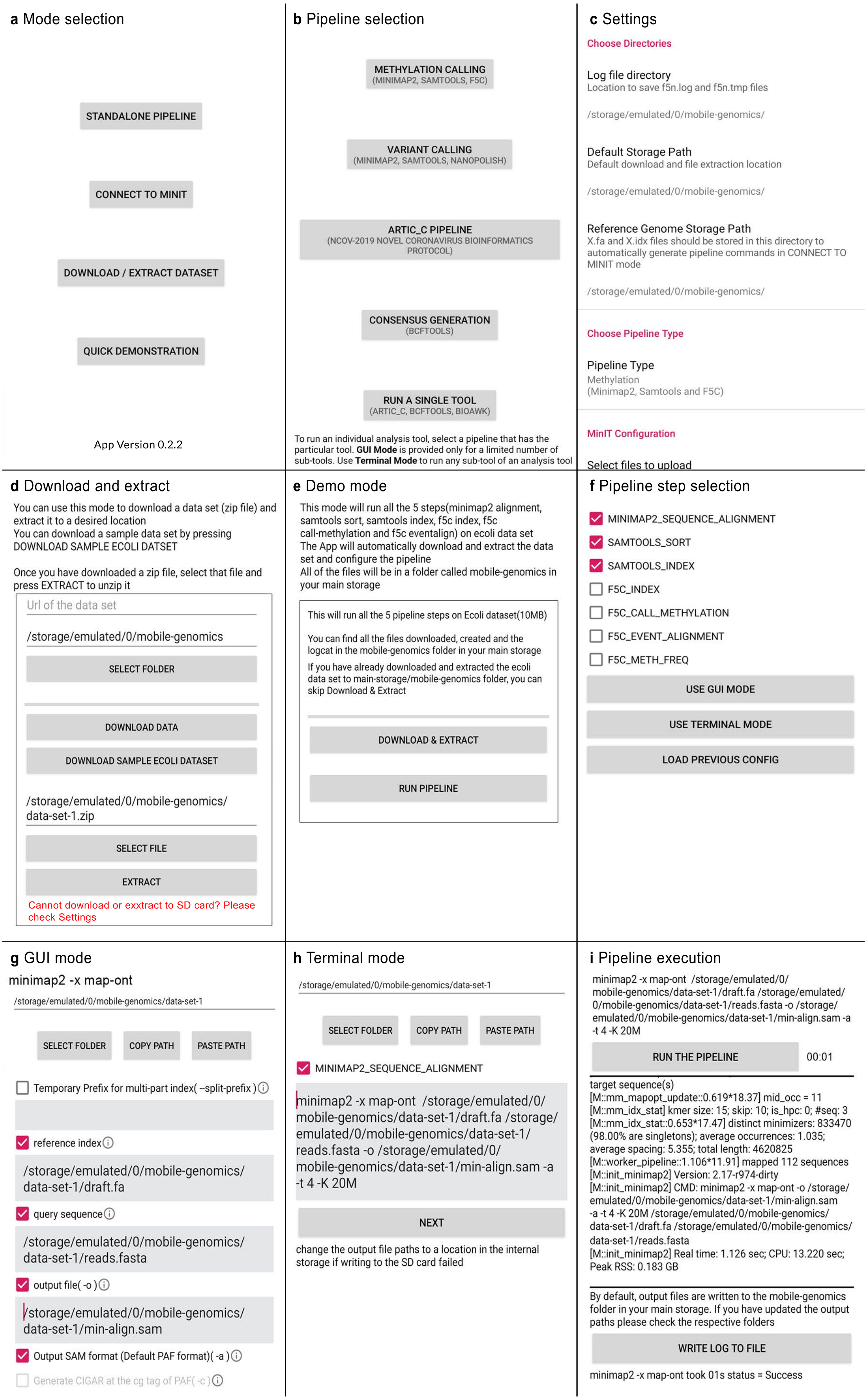

Fig. S4. Example screenshots illustrating basic Genopo user experience: (**a**) Homepage. (**b**) Pipeline selection. (**c**) Set *Genopo* Settings. (**d**) Download and extract files. (**e**) Run an example pipeline. (**f**) Select pipeline steps. (**g**) Configure pipeline steps using GUI. (**h**) Configure pipeline steps using terminal environment. (**i**) Pipeline execution.

SUPPLEMENTARY REFERENCES

1. Li, H. Minimap2: pairwise alignment for nucleotide sequences. *Bioinformatics* **34,** 3094–3100 (2018).
2. Li, H. A statistical framework for SNP calling, mutation discovery, association mapping and population genetical parameter estimation from sequencing data. *Bioinformatics* **27,** 2987–2993 (2011).
3. Simpson, J. T. *et al.* Detecting DNA cytosine methylation using nanopore sequencing. *Nat Methods* **14,** 407–410 (2017).
4. Quinlan, A. R. & Hall, I. M. BEDTools: a flexible suite of utilities for comparing genomic features. *Bioinformatics* **26,** 841–842 (2010).
5. Gamaarachchi, H., Parameswaran, S. & Smith, M. A. Featherweight long read alignment using partitioned reference indexes. *Sci Rep* **9,** 4318 (2019).
6. Gamaarachchi, H. *et al.* GPU accelerated adaptive banded event alignment for rapid comparative nanopore signal analysis. *BMC Bioinformatics* **21,** 343 (2020).
