# Supplementary material for: Genopo: a nanopore sequencing analysis toolkit for portable Android devices
Source: Commun Biol. 2020 Sep 29;3:538. doi: 10.1038/s42003-020-01270-z (PMC7524736; doi:10.1038/s42003-020-01270-z)
Supplement: Supplementary file 2 — Description of Additional Supplementary Files [file 42003_2020_1270_MOESM2_ESM.pdf]

Description of Additional Supplementary Files

File Name: Supplementary Data 1
Description: Detailed run-time information for SARS-CoV-2 genome analysis

File Name: Supplementary Data 2
Description: Detailed run-time information for NA12878 methylation calling analysis.
